# Long-term outcomes of total ureterectomy with ileal-ureteral substitution treatment for ureteral cancer: a single-center experience

**DOI:** 10.1186/s12894-018-0389-5

**Published:** 2018-08-31

**Authors:** Yin-Chien Ou, Che-Yuan Hu, Hong-Lin Cheng, Wen-Horng Yang

**Affiliations:** Department of Urology, National Cheng Kung University Hospital, No.138, Sheng Li Road, Tainan, 704 Taiwan, Republic of China

**Keywords:** Upper urinary tract urothelial carcinoma, Total ureterectomy, Ileum, Reconstructive surgical procedures

## Abstract

**Background:**

To explore the feasibility and long-term outcomes of renal preservation in a retrospective cohort of patients with ureteral urothelial carcinoma undergoing total ureterectomy with ileal-ureteral substitution.

**Methods:**

A retrospective review of the data from patients treated with total ureterectomy with ileal-ureteral substitution from 1988 to 2016 was performed. The pre-operative oncological status, long-term oncological outcome, long-term renal functional outcome, early and late complications were analyzed.

**Results:**

A total of eight patients with a median age of 70 years were included. The median follow-up time was 109 months. Six patients had multi-focal tumor involvement over the target ureter, and six patients had bilateral upper tract involvement. Only one patient encountered the upper-tract recurrence. The 2 and 5-year cancer-specific survival rates were 87.5 and 75.0%, respectively. The renal function was well-preserved in most patients, with only one patient needed life-long postoperative hemodialysis. Five patients experienced early complications and four patients experienced late complications. No perioperative mortality happened.

**Conclusions:**

A total ureterectomy with an ileal-ureteral substitution is feasible for treating ureteral urothelial carcinoma when a renal-sparing procedure is indicated. It provides good long-term oncological outcomes over the upper tract, and it also preserves the renal function.

## Background

Upper-tract urothelial carcinoma (UTUC) is a high-incidence disease in southern Taiwan. It has been associated with arsenic exposure and consumption of Chinese herbal products that contain aristolochic acid [1, 2]. Radical nephroureterectomy with bladder cuff excision provides durable oncological control, and remains the gold standard for treating UTUC [3]. However, kidney sparing surgery may take an important role in the arsenic or aristolochic acid endemic area, because the incidence of bilateral upper tract involvement was reported around 8–10% in the geographical region of aristolochic acid nephropathy [4, 5]. Also, the recurrence of UCs in the contralateral upper tract was reported to occur in 2–6% [6, 7]. Since the incidence of ureteral urothelial carcinoma (UC) is twice higher than the renal pelvic UC over the endemic area of arsenic exposure [2], kidney sparing surgeries are now considered reasonable alternatives in selected patients with chronic renal insufficiency, a solitary functional kidney, synchronous bilateral UTUCs or high risk of contralateral upper urinary tract recurrence.

Some researchers [8–10] have reported technical feasibility and good oncologic outcome in patients with ureteral UCs, using kidney sparing procedures such as endoscopic ablation and segmental ureterectomy (SU). However, endoscopic management may carry risks of under-staging, under-grading, or increased risk of recurrence, where SU may not be feasible in multifocal disease or in the case of long segment of tumor involvement [8]. Both procedures also encounter certain risk of recurrence on the remaining ureter [11, 12].

Total ureterectomy provides a maximal resection of the diseased ureter from uretero-pelvic junction to bladder cuff, which is also a reasonable alternative for patients with multifocal ureteral disease. Some case series [13–15] have reported that the combination of a total ureterectomy and renal auto-transplantation with a pyelocystostomy eliminates a potential multifocal ureteral UCs over the entire ureter. However, graft function and vascular complications are the major concerns of this procedure [13, 16–18]. Ileal-ureteral substitution was designed to bridge a huge ureteral defect, and the safety was showed in some studies [19–21]. However, only small case series and case reports discussed about the combination of total ureterectomy with ileal-ureteral substitution in treating ureteral UC [12, 22, 23]. Besides, these studies did not focus on the long-term functional and oncological outcome specifically for this procedure. We present a single-center’s experience of the total ureterectomy with ileal-ureteral substitution for ureteral UC patients in southern Taiwan over the last two decades after a long-term follow up.

## Methods

### Study design

We retrospectively reviewed the medical records of all patients who had a total ureterectomy with an ileal-ureteral substitution for ureteral UC at our center from January 1988 through December 2016. Approval from our hospital’s Institutional Review Board had been obtained before the commencement of the study (IRB number: A-ER-105-059). The patients’ underlying diseases, baseline renal function, surgical planning, pathology reports, early complications, late complications, long-term renal function, and the oncological outcomes were collected. The baseline renal function was evaluated using serum creatinine levels 1 day before surgery. The estimated glomerular filtration rate (eGFR) was calculated using the Modification of Diet in Renal Disease equation, and the patients were classified into different stages of chronic kidney disease (CKD). The pathology reports for these patients were reviewed by a single pathologist, and were translated into clinical grading and staging based on the *AJCC Cancer Staging Manual, 7th edition*. Complications were taken into consideration only if they were related to the surgery. Early complications were defined as those that occurred within 30 days after surgery, and late complications were defined as those at least 30 days after surgery. Early complications were rated major or minor based on the Clavien-Dindo classification of surgical complications [24]. Upper tract recurrence was defined as any documented radiographic or pathological evidence of cancer recurrence inside the ileal ureter, the ipsilateral renal pelvis, or over the surgical field. Bladder recurrence and distant metastasis data were also collected for oncological outcomes. Overall recurrence was defined as the combination of local recurrence, bladder recurrence, and any detectable distant metastasis.

### Surgical procedures

All patients underwent surgeries with similar technique by two surgeons. The main surgical steps were described as follows. A midline incision was made to approach into the abdominal cavity. Enter the retroperitoneal cavity and wide dissection to expose the renal pelvis and ureter. En bloc resection from ureteropelvic junction till bladder cuff was performed to remove the pathologic ureter. An ileal segment about 20 to 25 cm was harvested at least 15 cm away from the ileocecal valve. The ileal graft was lying in the isoperistaltic direction, with end-to-end pyelo-ileal anastomosis, and end-to-side reimplantation into the posterior wall of the urinary bladder. A double-J ureteral stent was placed before completion of the anastomosis. Retroperitonization of the reconstructed ileal segment was done to avoid urine leakage or tumor seeding into peritoneal cavity. No anti-reflux system was performed due to the concern of any obstructive condition in the solitary kidney patients. A suction drain is positioned in retroperitoneum close to anastomotic sites. A Foley catheter was inserted in the bladder and left for at least 1 week postoperatively.

### Follow-up for outcomes

All patients were regularly followed-up in our hospital after the total ureterectomy with ileal-ureteral substitution. Chest X-ray, renal ultrasonography, intravenous urography, and abdominal computed tomography imaging were done at regular intervals to evaluate any evidence of anastomotic obstruction, local recurrence, or distant metastasis. All patients underwent cystoscopy and semi-rigid ureteroscopy evaluation every 3 months during the first 2 years, every 6 months for the following 2 years, and then annually. Renal function was closely monitored based on serum creatinine levels. Metabolic derangements were monitored via biochemical blood tests including serum bicarbonate and chloride levels.

### Statistical analysis

SPSS 17.0 (SPSS Inc., Chicago, IL, USA) was used for all statistical analyses. The data are presented as median values due to small numbers and wide ranges.

## Results

### Population

Eight patients, including two men and six women, underwent total ureterectomy with ileal-ureteral substitution for ureteral UC. The median age for these patients were 70 years (range: 37–78) when they received the surgery. Substantial follow-up data were available for all patients, with a median follow-up period of 109 months (range: 23–167). All the resected ureters, six from right side and two from left side, were confirmed to have ureteral UCs. Four of them had pTa tumors, one had a pT1 tumor, and another three had pT2 tumors. All the surgical margins were free from tumor involvement.

Two patients (patient #1 and #8) presented with bilateral synchronous UTUCs. For them, a nephroureterectomy with bladder cuff excision for one side and a total ureterectomy with ileal-ureteral substitution for the other side were done as a single surgery. Patient #8 also had a pT2 bladder UC near left ureteral orifice, which was grossly removed by transurethral resection prior to this surgery. She insisted to preserve her bladder, and refused the suggestion of radical cystectomy. Therefore, a concomitant partial cystectomy was done along with the left nephroureterectomy, and the final pathology report revealed a pT3 disease with free surgical margin for her bladder specimen.

Four patients (patient #4, #5, #6, and #7) presented with bilateral asynchronous UTUCs. They all had received nephroureterectomy with bladder cuff excision for the contralateral kidney, and had lived with a solitary functional kidney before receiving the total ureterectomy with ileal-ureteral substitution. Beside the ureteral tumor, Patient #6 also presented with an upper calyceal tumor. He insisted to keep him away from hemodialysis as long as possible, and therefore a concomitant partial nephrectomy was performed for his upper calyceal lesion.

Two patients (patient #2 and #3) presented with unilateral UTUC. Patient #2 had lived with a solitary functional kidney due to a prior radical nephrectomy for contralateral renal cell carcinoma. Patient #3 had multifocal ureteral UC, but he favored kidney-sparing surgery after fully understanding the pros and cons of the procedure.

Four patients had previous history of bladder UC. All of them had received appropriate management and follow-up before this surgery. No patient had evidence of lymph node involvement according to pre-operative abdominal computed tomography scan. The clinical characteristics and oncological status of these eight patients are showed in Table 1.

**Table 1:** The clinical characteristics and oncological status of the patients

Patient No.	Age (years)	Sex	OP side	UC involvement of the target ureter (Sections involved)	Pathology of the ureter (stage/grade)	Extension of UTUC	Previous history of bladder UC
1	75–80	F	Rt	Multifocal (upper and lower)	pTa/High	Bilateral synchronous	
2	70–75	F	Rt	Multifocal (upper to lower)	pT2/High	Unilateral	
3	35–40	M	Lt	Multifocal (upper to lower)	pTa/Low	Unilateral	
4	70–75	F	Rt	Unifocal (middle)	pTa/High	Bilateral asynchronous	V
5	70–75	F	Rt	Multifocal (upper)	pT1/Low	Bilateral asynchronous	V
6	55–60	M	Lt	Multifocal (upper to lower)	pTa/High	Bilateral asynchronous	V
7	55–60	F	Rt	Unifocal (lower)	pT2/High	Bilateral asynchronous	
8	70–75	F	Rt	Multifocal (middle to lower)	pT2/High	Bilateral synchronous	V

*OP* operation, *UC* urothelial carcinoma, *UTUC* upper tract urothelial carcinoma

### Early and late complications

Four major (Clavien grade III–IV) and two minor (Clavien grade I–II) early complications developed in four patients. Two of the four major complications required a secondary surgical exploration for the leakage of pyelo-ileal anastomosis (patients #2 and #6), and the other two required temporary hemodialysis because of blood clot obstructions in the renal pelvis (patients #4 and #6). The two minor complications, including one self-limited urine leakage (patient #3) and one urinary tract infection (patient #2), were conservatively treated. There was no perioperative mortality.

Four patients had late complications throughout the follow-up period. Patient #6 had recurrent episodes of gross hematuria, and deteriorating renal function that finally required long-term hemodialysis. The patient’s remaining kidney and ileal-ureter were resected 1 year after the total ureterectomy. Both the resected kidney and the ileal-ureter were free of tumor recurrence. Patients #5 and #7 had recurrent urinary tract infections, and were managed with oral antibiotics on an outpatient basis. Long-term prophylaxis antibiotics were not necessary in both patients. Hyperchloremic metabolic acidosis was diagnosed in patient #1 and #5, with serum bicarbonate levels less than 20 mmol/l and serum chloride levels exceeding 107 mmol/l. They both had adequate respiratory compensation, and no medication was given throughout the follow-up period. The complications of each patient are listed in Table 2. The Clavien classifications and managements for the complications are showed in Table 3.

**Table 2:** The oncological outcome, renal function, and complications of the patients

Patient No.	F/u period (mo)	Time to recurrence (mo)	Site of recurrence	Pre-operative CKD stage (eGFR, mL/min)	Long-term CKD stage (eGFR, mL/min)	Early complications	Late complications
1	137	Nil		IV (22.94)	IV (21.27)		Metabolic acidosis
2	41	12	Bladder	III (39.51)	II (65.23)	Urine leakage	
3^a^	157	Nil		II (80.06)	I (106.14)	Urine leakage	
4^a^	167	Nil		III (58.26)	III (36.95)	Acute kidney injury	
5^a^	81	Nil		IV (27.21)	V (10.88)		Recurrent UTI Metabolic acidosis
6	141	23	Bladder	V (12.21)	H/D	Urine leakage Acute kidney injury	Recurrent hematuria
7^b^	56	42	Upper tract^c^	IV (15.81)	III (32.26)		Recurrent UTI
8^b^	23	12	Bladder	III (51.47)	III (57.14)	UTI	

*F/u* follow-up, *CKD* chronic kidney disease, *H/D* Hemodialysis, *UTI* urinary tract infection, *eGFR* estimated glomerular filtration rate

^a^Patients who are still alive

^b^Patients who had distant metastasis during follow-up

^c^Urothelial carcinoma recurrence was found over pelvic-ileal anastomosis

**Table 3:** The early and late complications, the Clavien-Dindo classification, and subsequent managements

Complications	Patients (*n*)	Management	Clavien-Dindo Classification
Early complications			
Self-limited urinary leakage	1	Conservative treatment	I
Urinary tract infection	1	Medical treatment	II
Leakage of anastomosis	2	Explore laparotomy	III
Acute kidney injury	2	Emergency hemodialysis	IV
Late complications			
Recurrent hematuria	1	Resection of the kidney and ileal ureter (after hemodialysis)	
Recurrent urinary tract infection	2	Medical treatment	
Mild hyperchloremic metabolic acidosis	2	No need of treatment	

### Long-term outcome of renal function

The baseline renal function of our patients included one with CKD stage II, three with CKD stage III, three with CKD stage IV, and one with CKD stage V. At the end of the follow-up, renal function improved in three patients, remained stable in three patients, and deteriorated in two patients. Only patient #6 required long-term hemodialysis. The baseline and long-term renal functions for each patient are listed in Table 2.

### Oncological outcome

Seven patients showed no evidence of upper tract recurrence on follow-up images. Patient #7 developed pyelo-ileal anastomotic stenosis 42 months after her ureterectomy. She subsequently underwent a pyeloplasty, and UC recurrence over the stenotic site was confirmed. She was the only patient with upper tract recurrence after the total ureterectomy with the ileal-ureteral substitution. Regular cystoscopy examinations showed that three patients had bladder UC recurrence; two of them had a previous history of bladder UC.

Two patients had distant metastasis. Patient #7 had an upper tract recurrence, and eventually died of UC with liver metastasis 56 months after the surgery. Patient #8 had a bladder recurrence during the follow-up, and she died of UC with bone metastasis 23 months after the surgery. The 5-year upper tract RFS rate were 87.5%. The 2 and 5-year recurrence-free survival (RFS) rates were 62.5 and 50%, respectively, and the 2 and 5-year cancer-specific survival (CSS) rates were 87.5 and 75.0%, respectively. Time to recurrence and location of recurrence for each patient are listed in Table 2.

## Discussion

Multifocal occurrence and frequent recurrence are well-known features of UTUC. Treating UTUC with radical nephroureterectomy and bladder cuff excision provides optimal tumor control and is now accepted as the gold standard [3]. However, resecting a kidney will cause excessive loss of renal function, which increases the risks of death and cardiovascular events [25]. Moreover, health-related quality of life progressively declines with more advanced stages of CKD [26]. Kidney-sparing procedures were designed to provide adequate local tumor control while preserving the kidney, and they are especially important in patients who will require dialysis if they receive nephroureterectomy. Five of our patients already lived with a solitary functional kidney preoperatively, and another two had synchronous bilateral diseases. For them, hemodialysis would have been inevitable without kidney-sparing procedures.

Several kidney-sparing procedures have been developed and evaluated for their pros and cons for treating UTUCs. Endoscopic management provides survival outcomes equivalent to that of a nephroureterectomy only in well selected patients with low-grade tumors [9]. Five-year CSS rates for SU were reported comparable to those of nephroureterectomy only in patients without proximal, long segment, or multifocal tumors [10]. Six patients in our series presented with multifocal diseases within the target ureter. Besides, the pathology report revealed that six patients had high grade tumors and five patients have a stage higher than Ta. Endoscopic management or SU are certainly not a good choice for these patients.

Total ureterectomy with renal autotransplantation has the advantage of complete resection for the pathological ureter. Cheng et al. [13] also reported the benefit of transurethral resection directly through the pyelocystostomy for renal pelvis recurrence after autotransplantation. However, this procedure also carries certain surgical risks. Delayed graft function and vascular complications including pseudoaneurysm, thrombosis, arterial stenosis, and hemorrhage have been reported [13, 16, 17]. Eisenberg et al. [18] concluded that renal autotransplantation has a higher rate of vascular complications than does allotransplantation. Seven of our patients had a baseline CKD stage III or above, and five of them were aged 70 or over. The complications that mentioned above might be catastrophic for them. Besides, the pyelocystostomy might create a route for ascending tumor seeding from the bladder to the renal pelvis, which, in turn, might compromise the oncological outcome and renal function preservation. Four patients in our series had a previous history of bladder UC, we thought it was not appropriate to link the renal pelvis directly to the bladder for these patients.

Using ileal-ureteral substitution for ureteral reconstruction was first described by Shoemaker in 1906, and was most commonly used for ureteral stricture after genitourinary surgery [22]. Other common indications include iatrogenic ureteral injury during a urological procedure, retroperitoneal fibrosis, and recurrent ureteropelvic junction obstruction [19–21]. The long-term safety for this procedure was well-discussed in previous studies [19–21]. A total ureterectomy with ileal-ureteral substitution not only provides maximal ureteral excision as wide as in renal autotransplantation, but avoids the devastating vascular complications. According to our review, only a few studies [12, 22, 23] used the total ureterectomy and ileal ureteral-substitution to treat ureteral UC. The present study, which focuses on the outcome of total ureterectomy with ileal-ureteral substitution in treating ureteral UCs, has the largest sample and the longest follow-up.

Most of the patients in our series had multifocal, invasive, and high grade UCs over their target ureters. Besides, six patients had bilateral upper tract diseases, and four of them also had bladder involvement. Our study presents a good oncological outcome for these patients with a median follow-up period of 109 months. Only one patients had an upper-tract recurrence over the pelvic-ileal anastomotic site 42 months postoperatively. Three patients, two with a history of bladder UC before, had bladder recurrence. The 2-year RFS and CSS rates were 62.5 and 87.5%, respectively, and the 5-year rates were 50.0 and 75.0%, respectively. Distant metastasis occurred in two patients, however one of whom (patient #8) had a pT3 bladder UC at the time of her total ureterectomy. Gadzinski et al. [9] reported excellent survival outcomes for endoscopic management in well-selected patients. The 5-year CSS rate for low-grade tumor was 100%, and for high-grade tumor was 85.7% in their group. However, Raymundo et al. [8] reported a 32% of under-staging and a 43% of upper tract recurrence in endoscopically managed patients. For properly selected patients treated with SU, the 2 and 5-year CSS rates were 92.2 and 86.8%, respectively [10], and the 5-year RFS rate (combining upper tract and bladder recurrence) was 37% [27]. Hung et al. [28] reported that the bladder recurrence, upper tract recurrence, and distant metastasis rates after SU were 34.2, 14.3, and 8.6%, respectively. Our literature review showed only some small series without long-term survival outcomes for UTUC treated with total ureterectomy and renal auto-transplantation. In addition to the overall recurrence and survival outcomes, we focused on upper tract recurrence because subsequent treatment is much more complicated than it is for bladder recurrence. Total ureterectomy provided excellent local oncological control, that seven of our eight patients were upper tract recurrence-free for 5 years. However, the urothelium remaining over the ipsilateral renal pelvis still has some risk of cancer recurrence, and this is the limitation for the procedure. Detailed and scheduled image follow-ups to detect any abnormal filling defect or anastomotic stenosis is extremely important for these patients.

Besides oncological control, maintaining residual renal function is the core concept of using kidney-sparing procedures to treat ureteral UCs in patients with a solitary kidney, bilateral tumors, or poor renal function. Most studies that focus on endoscopic management or SU provide no information about the maintenance of renal function. A recent review article reported that 74.7% of patients treated with ileal-ureteral substitution had reduced or stable postoperative serum creatinine levels [22]. In our patients, the ileal ureter was used to repair the huge defect between the renal pelvis and the urinary bladder after a total ureterectomy. All of our patients had stage II or higher baseline CKD. Six patients had stable or improved renal function during the long-term follow-up. We thought the improvement might be related to the relatively broad lumen after substitution of ileum to the pathologic ureter. Only one patient required postoperative life-long dialysis. He had a solitary kidney with CKD stage V at baseline, and he underwent a concomitant partial nephrectomy for upper calyceal UC. He underwent temporary hemodialysis because of a perioperative blood clot obstruction in his renal pelvis, and thereafter his renal function gradually deteriorated.

Using the ileum to replace the ureter carries certain risks: anastomotic complications, bowel complications, and wound infection [19–22]. Our patients had four major and two minor early complications but no surgically related mortality. In the literature, anastomotic urine leakage was the most common perioperative complication, and recurrent urinary tract infection was the most frequently mentioned long-term complication [19–22]. We present a similar result that two recurrent urinary tract infections during the long-term follow up. Several studies had mentioned about the hyperchloremic metabolic acidosis after the ileal ureteral substitution [19, 20, 22, 29]. The metabolic disorders seem more prevalent in patients with poor renal function, using excessive size of the ileal graft, or increasing the exposure of the bowel mucosa to urine. All our patients had relatively broad lumen after substitution of ileum, and they underwent regular image follow up to ensure the patency and fast flow of the urine from the renal pelvis down into the bladder. We propose that the urine is exposed to the bowel mucosa in a very limited time span. Therefore, only two patients in our series were found to have metabolic acidosis. Both of them tolerated the condition well under adequate respiratory compensation. This results implied that ileal interposition in individuals with poor preoperative renal function may still be feasible when the substitution is made only to the ureter, and the bladder is remained as a reservoir of urine.

This study has certain limitations. First, this study is retrospectively designed without a control group. The unique preoperative characteristics of these patients such as multifocal ureteral UCs, simultaneous or non-simultaneous bilateral upper tract involvement, high grade disease which is not suitable for conventional nephroureterectomy or other renal preservation surgeries, make it difficult to provide a matched case-control study. Second, statistical analysis is not feasible in a relatively small sample size. Nevertheless, we introduce a safe renal conservation surgery with good control of oncological outcome in such a special cohort of patients. Further research in a multicenter prospective randomized controlled design may help to draw a more explicit conclusion.

## Conclusions

Total ureterectomy with ileal ureteral substitution is a feasible choice of renal conservation surgery in treating ureteral UC. It is especially indicated in CKD patients having multi-focal ureteral UCs or long segment ureteral UC, when traditional renal conservation surgeries cannot have adequate tumor control. In our experience, this method not only provided maximal local excision of the involved ureter with a good long-term oncological outcome, but also preserved the residual renal function without remarkable complications.

## Abbreviations

CKD: Chronic kidney disease
CSS: Cancer-specific survival
eGFR: estimated glomerular filtration rate
RFS: Recurrence-free survival
SU: Segmental ureterectomy
UC: Urothelial carcinoma
UTUC: Upper-tract urothelial carcinoma

## References

[1] Chen CH, Dickman KG, Moriya M, Zavadil J, Sidorenko VS, Edwards KL (2012). Aristolochic acid-associated urothelial cancer in Taiwan. Proc Natl Acad Sci U S A.

[2] Yang MH, Chen KK, Yen CC, Wang WS, Chang YH, Huang WJ (2002). Unusually high incidence of upper urinary tract urothelial carcinoma in Taiwan. Urology.

[3] Margulis V, Shariat SF, Matin SF, Kamat AM, Zigeuner R, Kikuchi E (2009). Outcomes of radical nephroureterectomy: a series from the upper tract urothelial carcinoma collaboration. Cancer.

[4] Dragicevic D, Djokic M, Pekmezovic T, Micic S, Hadzi-Djokic J, Vuksanovic A (2007). Survival of patients with transitional cell carcinoma of the ureter and renal pelvis in Balkan endemic nephropathy and non-endemic areas of Serbia. BJU Int.

[5] Colin P, Koenig P, Ouzzane A, Berthon N, Villers A, Biserte J (2009). Environmental factors involved in carcinogenesis of urothelial cell carcinomas of the upper urinary tract. BJU Int.

[6] Novara G, De Marco V, Dalpiaz O, Galfano A, Bouygues V, Gardiman M (2009). Independent predictors of contralateral metachronous upper urinary tract transitional cell carcinoma after nephroureterectomy: multi-institutional dataset from three European centers. Int J Urol.

[7] Li WM, Shen JT, Li CC, Ke HL, Wei YC, Wu WJ (2010). Oncologic outcomes following three different approaches to the distal ureter and bladder cuff in nephroureterectomy for primary upper urinary tract urothelial carcinoma. Eur Urol.

[8] Roupret M, Hupertan V, Traxer O, Loison G, Chartier-Kastler E, Conort P (2006). Comparison of open nephroureterectomy and ureteroscopic and percutaneous management of upper urinary tract transitional cell carcinoma. Urology.

[9] Gadzinski AJ, Roberts WW, Faerber GJ, Wolf JS (2010). Long-term outcomes of nephroureterectomy versus endoscopic management for upper tract urothelial carcinoma. J Urol.

[10] Jeldres C, Lughezzani G, Sun M, Isbarn H, Shariat SF, Budaus L (2010). Segmental ureterectomy can safely be performed in patients with transitional cell carcinoma of the ureter. J Urol.

[11] Suriano F, Brancato T (2014). Nephron-sparing Management of Upper Tract Urothelial Carcinoma. Rev Urol.

[12] Pedrosa JA, Masterson TA, Rice KR, Kaimakliotis HZ, Monn MF, Bihrle R (2015). Oncologic outcomes and prognostic impact of urothelial recurrences in patients undergoing segmental and total ureterectomy for upper tract urothelial carcinoma. Can Urol Assoc J.

[13] Cheng YT, Flechner SM, Chiang PH (2014). The role of laparoscopy-assisted renal autotransplantation in the treatment of primary ureteral tumor. Ann Surg Oncol.

[14] Pettersson S, Brynger H, Henriksson C, Johansson SL, Nilson AE, Ranch T (1984). Treatment of urothelial tumors of the upper urinary tract by nephroureterectomy, renal autotransplantation, and pyelocystostomy. Cancer.

[15] Holmang S, Johansson SL (2005). Tumours of the ureter and renal pelvis treated with resection and renal autotransplantation: a study with up to 20 years of follow-up. BJU Int.

[16] Orlic P, Vukas D, Drescik I, Ivancic A, Blecic G, Budiselic B (2003). Vascular complications after 725 kidney transplantations during 3 decades. Transplant Proc.

[17] Webster JC, Lemoine J, Seigne J, Lockhart J, Bowers V (2005). Renal autotransplantation for managing a short upper ureter or after ex vivo complex renovascular reconstruction. BJU Int.

[18] Eisenberg ML, Lee KL, Zumrutbas AE, Meng MV, Freise CE, Stoller ML (2008). Long-term outcomes and late complications of laparoscopic nephrectomy with renal autotransplantation. J Urol.

[19] Wolff B, Chartier-Kastler E, Mozer P, Haertig A, Bitker MO, Roupret M (2011). Long-term functional outcomes after ileal ureter substitution: a single-center experience. Urology.

[20] Verduyckt FJ, Heesakkers JP, Debruyne FM (2002). Long-term results of ileum interposition for ureteral obstruction. Eur Urol.

[21] Chung BI, Hamawy KJ, Zinman LN, Libertino JA (2006). The use of bowel for ureteral replacement for complex ureteral reconstruction: long-term results. J Urol.

[22] Armatys SA, Mellon MJ, Beck SD, Koch MO, Foster RS, Bihrle R. Use of ileum as ureteral replacement in urological reconstruction. J Urol. 2009;181(1):177–81. PubMed PMID: 19013597. Pubmed Central PMCID: PMC2667902. Epub 2008/11/18. eng 10.1016/j.juro.2008.09.019 PMC2667902 19013597

[23] Banerji JS, George AJ (2014). Total ureterectomy and ileal ureteric replacement for TCC ureter in a solitary kidney. Can Urol Assoc J.

[24] Dindo D, Demartines N, Clavien PA (2004). Classification of surgical complications: a new proposal with evaluation in a cohort of 6336 patients and results of a survey. Ann Surg.

[25] Go AS, Chertow GM, Fan D, McCulloch CE, Hsu CY (2004). Chronic kidney disease and the risks of death, cardiovascular events, and hospitalization. N Engl J Med.

[26] Mujais SK, Story K, Brouillette J, Takano T, Soroka S, Franek C (2009). Health-related quality of life in CKD patients: correlates and evolution over time. Clin J Am Soc Nephrol.

[27] Zincke H, Neves RJ (1984). Feasibility of conservative surgery for transitional cell cancer of the upper urinary tract. Urol Clin North Am.

[28] Hung SY, Yang WC, Luo HL, Hsu CC, Chen YT, Chuang YC (2014). Segmental ureterectomy does not compromise the oncologic outcome compared with nephroureterectomy for pure ureter cancer. Int Urol Nephrol.

[29] Shokeir AA (1997). Interposition of ileum in the ureter: a clinical study with long-term follow-up. Br J Urol.

